# The Comparison of the Surgical Outcome for the Full-Thickness Macular Hole with/without Lamellar Hole-Associated Epiretinal Proliferation

**DOI:** 10.1155/2017/9640756

**Published:** 2017-12-13

**Authors:** Yuri Ubukata, Hisanori Imai, Keiko Otsuka, Masaya Nishizaki, Rumiko Hara, Mamoru Uenishi, Atsushi Azumi, Makoto Nakamura

**Affiliations:** ^1^Department of Surgery-Related, Division of Ophthalmology, Kobe University Graduate School of Medicine, 7-5-2 Kusunoki-cho, Chuo-ku, Kobe 650-0017, Japan; ^2^Department of Ophthalmology, Kitaharima General Medical Center, 926-250 Ichiba-cho, Ono 675-1392, Japan; ^3^Department of Ophthalmology, Kakogawa Nishi Municipal Hospital, 384-1 HIratsu, Yoneda-cho, Kakogawa 675-0054, Japan; ^4^Department of Ophthalmology, Mitsubishi Kobe Hospital, 6-1-34 Wadamiya Street, Hyogo-ku, Kobe 652-0863, Japan; ^5^Department of Ophthalmology, Kobe Kaisei Hospital, 3-11-15 Shinoharakitamachi, Nada-ku, Kobe 657-0068, Japan

## Abstract

**Aims:**

To compare the surgical outcome of pars plana vitrectomy (PPV) for full-thickness macular hole (FTMH) with and without lamellar hole-associated epiretinal proliferation (LHEP).

**Methods:**

This retrospective study included 158 eyes of 158 patients with FTMH treated with PPV. The following variables were analyzed: sex, age, preoperative best corrected visual acuity (pre-BCVA), BCVA 6 months after the surgery (6M-BCVA), the axial length of eye, the minimum diameter of FTMH, the diameter of basal side of FTMH, postoperative continuity of subfoveal ellipsoid zone (EZ) and external limiting membrane (ELM), and the preoperative presence of LHEP.

**Results:**

Twenty-eight eyes had FTMH with LHEP and 130 without LHEP. The mean ± SD age (years) was 72.6 ± 7.9 and 68.6 ± 8.7, respectively (*p* = 0.02). 6M-BCVA was 0.38 ± 0.30 and 0.26 ± 0.25, respectively (*p* = 0.03). The diameter of basal side of FTMH (*μ*m) was 901.5 ± 404.9 and 658.9 ± 288.1, respectively (*p* = 0.00027). EZ was disrupted in 24 eyes and 63 eyes, respectively (*p* = 0.00071). ELM was disrupted in 15 eyes and 23 eyes, respectively (*p* = 0.00015). The FTMH diameters and the presence of LHEP were inversely correlated with the continuity of EZ and ELM.

**Conclusion:**

The preoperative appearance of LHEP could be one of the prognostic factor for the treatment of FTMH.

## 1. Introduction

Two distinct mechanisms have been reported for the full-thickness macular hole (FTMH) formation. A major mechanism is vitreomacular separation secondary to tangential vitreofoveal traction [1, 2]. On the other hand, the other is the separation of the fovea due to the contraction of epiretinal membrane (ERM) on the lamellar macular hole (LMH) [1, 3, 4]. It is well known that LMH frequently involves ERM [5]. It has recently been reported that 30.5–60% of LMH involve lamellar hole-associated epiretinal proliferation (LHEP) [6, 7], which is thickened yellowish-pigmented tissue [8], and that the presence of LHEP is inversely related to the integrity of photoreceptor and visual function in LMH cases [7, 9]. A recent study has demonstrated that the LHEP was present in 8–9.6% of FTMH cases as well [6, 7]. However, there is no comparative study which investigated whether the presence of the LHEP affects postoperative visual function and morphologic characteristics following transconjunctival sutureless pars plana vitrectomy (TSV) for FTMH. The purpose of this study was to compare the functional and morphologic outcomes of TSV for FTMH with LHEP in reference to FTHM without LHEP.

## 2. Patients and Methods

This multicenter retrospective comparative study reviewed all medical records of patients with idiopathic FTMH treated with 25-gauge or 27-gauge TSV from January 2010 to April 2015. This study was approved by the institutional review board in each institution and conformed to the tenets of the Declaration of Helsinki. 158 eyes of 158 consecutive patients were enrolled. Eyes with FTMH secondary to high myopia, defined as preoperative refractive error (spherical equivalent) greater than −6.0 diopters (D) in phakic eyes or axial length longer than 26 mm, a history of prior intraocular surgeries for vitreoretinal diseases, and with a postoperative follow-up period less than 6 months were excluded in this study. The following variables were analyzed: sex, age, preoperative best corrected visual acuity (pre-BCVA), BCVA 6 months after the surgery (6M-BCVA), the axial length of eye (EL), the minimum diameter of FTMH, the diameter of basal side of FTMH, the continuity of subfoveal ellipsoid zone (EZ) and external limiting membrane (ELM) 6 months after the surgery, and the preoperative presence or absence of LHEP. We did not include the presence or absence of ERM as a variable for the statistical analysis because ERM did not exist in all cases of FTMH without LHEP and exists in all cases of FTMH with LHEP in this study. The presence of FTMH and the continuity of EZ and ELM were judged by images obtained from commercially available spectral-domain optical coherence tomography (OCT, Spectralis HRA + OCT; Heidelberg Engineering, Heidelberg, Germany). The presence of LHEP was judged either by OCT images or intraoperative observation of yellowish pigment around the macular hole. The presence of ERM was also judged either by OCT images or intraoperative observation. The size of FTMH was manually measured using the software Caliper in OCT.

## 3. Statistical Methods

The chi-square test and Fisher's exact probability test for dichotomous variables and unpaired *t*-test for continuous variables were used to compare parameters. Canonical correlation analysis was used to evaluate the relationships between preoperative parameters (e.g., sex, age, pre-BCVA, EL, the minimum diameter of FTMH, the diameter of basal side of FTMH, and the presence of LHEP) and postoperative parameters (e.g., 6M-BCVA, the continuity of subfoveal EZ and ELM 6 months after the surgery). The Landolt decimal visual acuity was converted to logMAR for statistical analysis. All statistical analyses were conducted using the XLSTAT software (Addinsoft, New York, USA). Statistical significance was inferred for *p* < 0.05.

## 4. Surgical Procedures

Standard 25- or 27-gauge TSV with a wide-angle noncontact viewing system (Resight®; Carl Zeiss Meditec AG, Jena, Germany) was performed under sub-Tenon anesthesia by approximately 4 mL of 2% lidocaine using the Constellation Vision System (Alcon Laboratories Inc., Fort Worth, TX, USA) in all cases. Before vitrectomy, phacoemulsification and intraocular lens implantation (PEA + IOL) were performed using the same machine for all phakic eyes. Following the core vitrectomy, vitreous gel was visualized by the injection of triamcinolone acetonide (MaQaid, Wakamoto Pharmaceutical, Tokyo, Japan) during midperipheral vitrectomy. Posterior vitreous detachment was induced if it was not present. If present, LHEP was gently peeled together with ERM with a special care to avoid tearing it from the FTMH edge. If left, it was then trimmed using the vitreous cutter as much as possible. After that, indocyanine green (0.25%) solution was applied to stain internal limiting membrane (ILM) and was immediately removed by suction. ILM peeling for 2 disc diameters around the FTMH was performed using microforceps. Finally, the peripheral vitreous gel was shaved for 360° with scleral indentation under a wide-angle noncontact viewing system. Complete fluid-air exchange was done in the vitreous cavity. At the end of surgery, all eyes were flushed with 50 mL of mix of nonexpansile gas (20% SF6) to assure a complete exchange. Additional gas mixture was injected through the pars plana to adjust intraocular pressure (IOP) if necessary. Any sclerotomy sites that were found to leak at the end of the surgery were sutured with 8-0 vicryl suture. IOP was checked by tactile examination. Subconjunctival corticosteroids were injected, and antibiotic ointment was administered at the end of the surgical procedure.

## 5. Results

Table 1 summarizes patients' perioperative demographic data. Twenty-eight eyes had FTMH with LHEP (group A) and 130 without LHEP (group B). There were no significant differences in sex proportion, pre-BCVA, and EL between the two groups. The patients with LHEP were significantly older than those without LHEP. Although the minimum diameter of the FTMH was not different between the two groups, the diameter of basal side of FTMH in the eyes with LHEP was significantly larger compared with those without LHEP preoperatively. The eyes with LHEP showed significantly worse 6M-BCVA than those without LHEP. The former group had a higher frequency of the disrupted EZ and ELM 6 months after the surgery.

**Figure 1:**
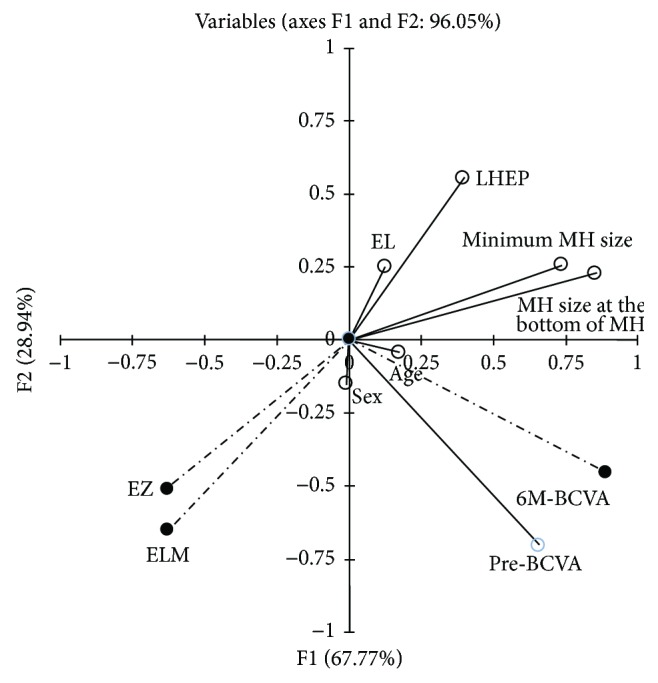
Canonical correlation analysis showing correlative relationships between postoperative and preoperative parameters. Preoperative visual acuity (pre-BCVA) was positively associated with visual acuity 6 months (6M-BCVA) after the surgery. The minimum MH diameter, the diameter of basal side of FTMH, and the presence of LHEP were inversely correlated with the postoperative continuity of ellipsoid zone (EZ) and external limiting membrane (ELM). The axial length of the eye (EL), age, and sex were not associated with any postoperative parameters. Postoperative parameters were shown with a broken line and black circle. Preoperative parameters were shown with a solid line and white circle.

We performed a canonical correlation analysis for the comparison between preoperative and postoperative parameters. We used the result of the first (F1) and the second (F2) canonical variate for the interpretation of data. The result of the third (F3) canonical variate was not used because it is not statistically significant (Table 2). Pre-BCVA was closely positively associated with 6M-BCVA. The minimum MH diameter, the diameter of basal side of FTMH, and the presence of LHEP were inversely correlated with the postoperative continuity of EZ and ELM. EL, age, and sex were not associated with any postoperative parameters (Figure 1).

## 6. Discussion

Previous reports stated that FTMH with LHEP accounts for 8-9.6% of all FTMH cases [6, 7]. In the present investigation, LHEP was present in 28 out of 158 eyes (17.7%), a higher rate than those in the past reports. This difference in results may be due to the disparity of definition of LHEP. The definition of LHEP was solely made by the OCT findings in the previous reports. On the other hand, Tsai et al. have reported that yellowish pigment around the FTMH was noticed in 10% of stage 2 FTMH and 13.6% of stage 4 FTMH, without OCT evidence of LHEP [10]. We included the case, which had yellowish pigment around the FTMH, as the presence of LHEP in this study. Collectively, these results may suggest that the specificity of OCT finding for the identification of LHEP is very high, but its sensitivity is not perfect. It is possible that the LHEP may be present in FTMH cases more than expected.

**Figure 2:**
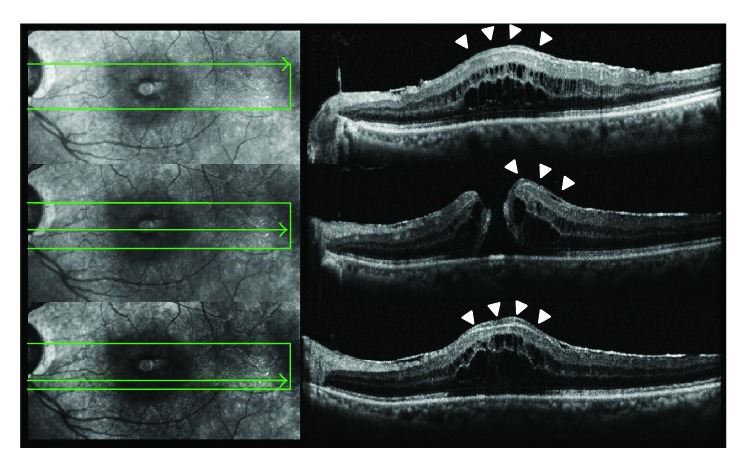
Typical OCT finding of FTMH with LHEP, which have a fluid cuff (white arrowheads). The minimum diameter of FTMH exists at the middle layer of the retina.

**Figure 3:**
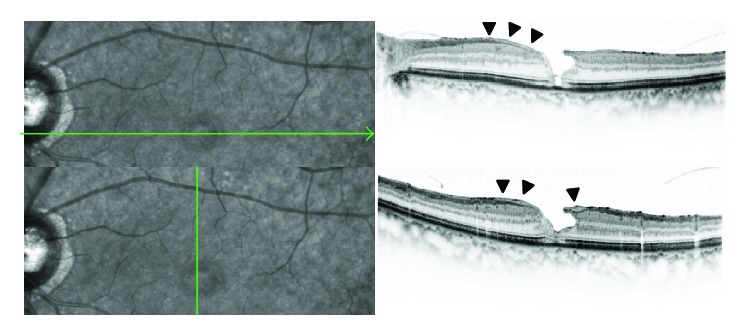
Typical OCT finding of FTMH with LHEP, which do not have a fluid cuff (black arrowheads). This type of FTMH has the widest inner opening and the narrowest base.

In this study, there was no difference in the smallest FTMH diameter between FTMH with and without LHEP. This result is at odd with previous reports [10, 11]. This discrepancy in results may be due to the existence of two different shapes in FTMH with LHEP. One is FTMH with a fluid cuff (Figure 2). The shape of FTMH in this type is similar to idiopathic FTMH, and the minimum diameter of FTMH exists at the middle layer of the retina. On the other hand, the other type of FTMH has the widest inner opening and the narrowest base without a fluid cuff (Figure 3). Thus, whereas minimum FTMH diameter and FTMH-base diameter differ from each other in cases with a fluid cuff, they are identical in cases without a fluid cuff. So, it could be said that “minimum size” is unstable and not suitable for the evaluation of the diameter of FTMH with LHEP. We measured the diameter of basal side of FTMH to evaluate its diameter at the same histological position in all cases. Our result showed that FTMH with LHEP featured a larger hole-based diameter and occurred in older patients than that observed in cases of FTMH without LHEP. Generally, the diameter of FTMH tends to get larger along with a prolonged period of FTMH and a progress of disease stage. We believe our result is reasonable because the disease duration in FTMH with LHEP is thought longer than idiopathic FTMH because many cases in this type of FTMH originate from LMH and had repeated episodes of spontaneous hole closure followed by subsequent reopening, which resulted in the formation of the larger FTMH [10, 11]. This is also indicated in our study from the finding that FTMH with LHEP was observed in older patients.

For this study, we used canonical correlation analysis, which is a method for exploring the relationships between two multivariate sets of variables, to explore the relationships between preoperative and postoperative factors. The result of canonical correlation analysis demonstrated strong positive association of preoperative BCVA with postoperative BCVA and inverse relationship of the FTMH diameter and the presence of LHEP with the postoperative photoreceptor integrity. Moreover, the eyes with LHEP showed the worse postoperative BCVA and higher frequency of disrupted continuity of EZ and ELM compared to those without LHEP. It has been already reported that the preoperative smaller FTMH diameter was a prognostic factor of better postoperative functional and structural outcomes [12]. The notable result in our study was the inverse relationships of the presence of LHEP with postoperative functional and morphological outcomes, which is consistent with the previous study [10]. These results strongly support the hypothesis that FTMH with LHEP develops first with processes of photoreceptor damage following LMH formation and later outer laminar dehiscence. The presence of LHEP may be a potential predictor of poor postoperative functional and morphological outcomes. Prospective, larger-scale study results are anticipated.

This study has potential limitations. First, because it is a retrospective study, there may have been a bias of patient selection. Another problem is that the sample size in each subgroup was relatively small.

In conclusions, we performed a comparative study of surgical outcomes for FTMH with and without LHEP. The preoperative presence of LHEP was inversely associated with the postoperative functional and morphological outcome. We believe the presence of LHEP might be one of the prognostic factors for the treatment of FTMH.

## Figures and Tables

**Table 1:** Demographic data for the patients.

Variables	LHEP+	LHEP−	*p* value
Number of eyes	28	130	
Sex, male/female	12/16	51/79	0.88
Age (years), mean ± SD	72.6 ± 7.9	68.6 ± 8.7	0.02
The axial length of the eye (mm), mean ± SD	24.6 ± 2.0	24.1 ± 1.7	0.16
Preoperative data			
Preoperative visual acuity (logMAR), mean ± SD	0.64 ± 0.24	0.65 ± 0.30	0.83
Minimum diameter of FTMH (*μ*m), mean ± SD	380.3 ± 237.2	314.4 ± 179.4	0.10
The diameter of basal side of FTMH (*μ*m), mean ± SD	901.5 ± 404.9	658.9 ± 288.1	0.00027
Postoperative data			
Visual acuity 6 months after the surgery (logMAR), mean ± SD	0.38 ± 0.30	0.26 ± 0.25	0.03
Ellipsoid zone, −/+	24/4	63/67	0.00071
External limiting membrane, −/+	15/13	23/107	0.00015

LHEP: lamellar hole-associated epiretinal proliferation; SD: standard deviation; FTMH: full-thickness macular hole.

**Table 2:** Results of the canonical correlation analysis for perioperative parameters.

	Canonical variate		
	F1	F2	F3
Postoperative data			
Visual acuity 6 months after the surgery	0.889	−0.455	−0.060
Ellipsoid zone	−0.629	−0.511	−0.586
External limiting membrane	−0.627	−0.652	0.425
Preoperative data			
Sex	−0.008	−0.153	−0.817
Age	0.174	−0.042	0.327
Preoperative visual acuity	0.659	−0.702	0.027
Minimum diameter of FTMH	0.736	0.257	−0.394
The diameter of basal side of FTMH	0.853	0.229	0.169
The axial length of eye	0.125	0.249	0.082
LHEP	0.396	0.556	0.071
Canonical correlation coefficient	0.693 (*p* < 0.0001)	0.453 (*p* = 0.0001)	0.153 (*p* = 0.611)

FTMH: full-thickness macular hole; LHEP: lamellar hole-associated epiretinal proliferation.

## References

[1] Yeh P. T., Chen T. C., Yang C. H. (2010). Formation of idiopathic macular hole—reappraisal. *Graefe's Archive for Clinical and Experimental Ophthalmology*.

[2] Gass J. D. M. (1988). Idiopathic senile macular hole: its early stages and pathogenesis. *Archives of Ophthalmology*.

[3] Allen A. W., Gass J. D. M. (1976). Contraction of a perifoveal epiretinal membrane simulating a macular hole. *American Journal of Ophthalmology*.

[4] Messmer E. M., Heidenkummer H. P., Kampik A. (1988). Ultrastructure of epiretinal membranes associated with macular holes. *Graefe's Archive for Clinical and Experimental Ophthalmology*.

[5] Androudi S., Stangos A., Brazitikos P. D. (2009). Lamellar macular holes: tomographic features and surgical outcome. *American Journal of Ophthalmology*.

[6] Pang C. E., Spaide R. F., Freund K. B. (2014). Epiretinal proliferation seen in association with lamellar macular holes: a distinct clinical entity. *Retina*.

[7] Itoh Y., Levison A. L., Kaiser P. K., Srivastava S. K., Singh R. P., Ehlers J. P. (2016). Prevalence and characteristics of hyporeflective preretinal tissue in vitreomacular interface disorders. *The British Journal of Ophthalmology*.

[8] Pang C. E., Maberley D. A., Freund K. B. (2016). Lamellar hole-associated epiretinal proliferation: a clinicopathologic correlation. *Retina*.

[9] Pang C. E., Spaide R. F., Freund K. B. (2015). Comparing functional and morphologic characteristics of lamellar macular holes with and without lamellar hole-associated epiretinal proliferation. *Retina*.

[10] Tsai C. Y., Hsieh Y. T., Yang C. M. (2016). Epiretinal membrane-induced full-thickness macular holes: the clinical features and surgical outcomes. *Retina*.

[11] Lai T. T., Chen S. N., Yang C. M. (2016). Epiretinal proliferation in lamellar macular holes and full-thickness macular holes: clinical and surgical findings. *Graefe's Archive for Clinical and Experimental Ophthalmology*.

[12] Ullrich S., Haritoglou C., Gass C., Schaumberger M., Ulbig M. W., Kampik A. (2002). Macular hole size as a prognostic factor in macular hole surgery. *The British Journal of Ophthalmology*.

